# Negative Pressure Wound Therapy as an Adjunct to Skin Grafting in Traumatic Injuries

**DOI:** 10.1590/1413-785220263401e292923

**Published:** 2026-03-30

**Authors:** Cleyton Chaves da Rocha, Ricardo Lavorato Filizzola, Luiz Sorrenti, Álvaro Baik Cho, Carlos Henrique Vieira Ferreira, Leandro Yoshinobu Kiyohara, Eduardo Romera Alves de Souza, Carina Rocha da Silva

**Affiliations:** 1 Faculdade de Medicina do ABC, Hospital Estadual Mario Covas, Grupo de Cirurgia da Mao e Microcirurgia, Sao Paulo, SP, Brazil.

**Keywords:** Skin Transplantation, Patient Isolators, Low Cost Technology, Wounds and Injuries, Enxertia de Pele, Isoladores de Pacientes, Tecnologia de Baixo Custo, Ferimentos e Lesões

## Abstract

**Introduction:**

Wounds are a challenge for health professionals and services, prolonging hospital stays and increasing morbidity and mortality rates. Wounds occur due to post-traumatic tissue loss, which is often difficult to resolve and is complicated by infections, local vascular impairment, and systemic diseases. To demonstrate the effectiveness of negative pressure therapy (NPT) using the vacuum gauge of the hospital gas pipeline and low-cost materials in the immediate postoperative period. To use resources available in daily hospital practice to create a low-cost vacuum dressing. To describe the equipment, preparation, and technique involved in low-cost NPT treatment as an adjuvant in the repair of lesions with indication for skin grafting.

**Methodology:**

Prospective, interventional, randomized clinical study with a non-inferiority design.

**Results:**

Fifteen patients were included at the end of the study, with complete follow-up until complete healing. The mean age of the selected patients was 38.27 years. Regarding gender, 93.3% were male and 6.7% were female.

**Conclusion:**

Thus, the results suggested that the use of low-cost NPT on skin grafts is a valuable, safe, easily reproducible and low-morbidity tool for surgeons technical arsenal. ***Level of Evidence: IV; Case Series***.

## RESUMO

**Introdução:**

As feridas constituem um desafio aos profissionais e serviços de saúde, prolongam o tempo de hospitalização e aumentam a taxa de morbimortalidade. As feridas ocorrem devido perdas teciduais pós trauma, muitas vezes de difícil resolução, complicadas por infecções, comprometimento vascular local, doenças sistêmicas.

**Objetivos:**

Demonstrar a efetividade do uso da terapia por pressão negativa (TPN) utilizando o vacuômetro da régua de gases hospitalares e materiais de baixo custo no pós-operatório imediato. Utilizar recursos disponíveis no cotidiano hospitalar para confeccionar um curativo a vácuo de baixo custo. Descrever os equipamentos, a preparação e a técnica envolvidos no tratamento de TPN de baixo custo como adjuvante na reparação de lesões com indicação de enxertia de pele.

**Metodologia:**

Estudo clínico prospectivo, intervencionista, randomizado com desenho de não inferioridade.

**Resultados:**

Foram incluídos 15 pacientes ao final do estudo, com acompanhamento completo até a cicatrização total. A média de idade dos pacientes selecionados foi de 38,27 anos. Quanto ao sexo tivemos 93,3% masculino e 6,7% feminino.

**Conclusão:**

Assim, os resultados sugerem que o uso da TPN de baixo custo sobre enxertos de pele constitui uma ferramenta de grande valia, segura, de fácil reprodutibilidade e associada a baixa morbidade no arsenal técnico dos cirurgiões. ***Nível de Evidência: IV; Série de Casos***.

## INTRODUCTION

The wounds constitute a challenge for health professionals and services, prolonging hospitalization time and increasing morbidity and mortality rates. Wounds occur due to tissue loss post-trauma, often difficult to resolve, complicated by infections, local vascular compromise, systemic diseases.^1^

In this study, we emphasize the use of skin grafts associated with negative pressure therapy (NPT). Apelqvist et al.^2^ describe that NPT has the ability to adapt to the contours of the wound bed, promoting the approximation of the edges and retraction of the lesion, mechanical stress on the wound edges, improving microcirculation and tissue perfusion, has an anti-edematous effect, leading to angiogenesis and granulation tissue formation and reduction of inflammatory exudate.

This method also provides the same benefits to surgical wounds, favoring proper and aseptic closure over an extended period, without the need for frequent interventions.^2^

The skin graft is the transfer of skin tissue between areas of the body, does not carry a vascularized pedicle and depends on an adequate recipient bed. After incorporation, skin grafts provide wounds with protection against the environment, pathogens, temperature, and excessive water loss like normal skin.^3^

Grafts may present some short-term complications: seroma, hematoma, infection, shearing or traction; and long-term: contracture, aesthetic problems, pigment and texture differences between the grafted skin and the donor site.^3^

According to the surveys by Lima et al.^1^, the integration of NPT after grafting was significantly greater when compared to conventional dressings, reducing the risks of complications.

In addition to better graft integration, NPT reduces hematoma formation, promotes a shear-free environment, and optimizes integration. In these cases, NPT should always be administered in continuous mode.^1^

NPT has been approved since 1997 by the *Food and Drug Administration* (FDA) in the United States. In 2003, NPT was introduced in Brazil, and in 2008, the simplified vacuum dressing with national technology was registered by the University of São Paulo (USP).^4,5^

The study by Kamamoto,^4^ shows us that the costs related to NPT can be quite high. Through the method developed by USP, in which a valve was designed that, when connected to the hospital gas network, can control the pressure exerted on the sealed dressing, using gauze on the wound bed and sealing film. The result of the work was able to counter the cost-effectiveness of the similar method compared to the commercial method regarding the stimulation of healing (effectiveness), in addition to having an average cost of $15.15 compared to $872.59 spent on the commercial method. According to the authors, the dressing model developed by USP, called "adapted low cost", did not show lower efficiency compared to the commercial "gold standard" system to which it was compared.^4^

This work is justified by the need for discussion and implementation of effective and safe systems that reduce the disturbances caused by traumatic injuries. Also, the promotion of new studies for reflection on such a relevant topic for public health. In this context, it is important that the health team develops wound repair techniques that optimize the healing of devitalized tissues.

## OBJECTIVES

Demonstrate the effectiveness of using TPN with the vacuum meter of the hospital gas gauge and low-cost materials in the immediate postoperative period of definitive closure of skin grafting in patients due to trauma.

To use available resources in the hospital routine to create a low-cost vacuum dressing.

To describe the equipment, preparation, and technique involved in low-cost NPT treatment as an adjunct in the repair of injuries with indications for skin grafting.

## METHODOLOGY

Prospective, interventional, randomized clinical study with a non-inferiority design. After approval from the Ethics Research Committee of the ABC School of Medicine under number: 7,187,630, the selected patients signed the Informed Consent Form.

This study was conducted in a high-complexity State Teaching Hospital in the Greater ABC region, in the state of São Paulo. The population consisted of patients with upper or lower limb injuries with tissue loss sufficient to prevent primary closure of the injuries, subjected to soft tissue injury coverage with skin grafts.

Inclusion criteria were patients over 18 years old, victims of high-energy trauma with wounds requiring skin grafting. Exclusion criteria were coagulopathies, uncontrolled active bleeding, and refusal to participate.

**Figure 1:**
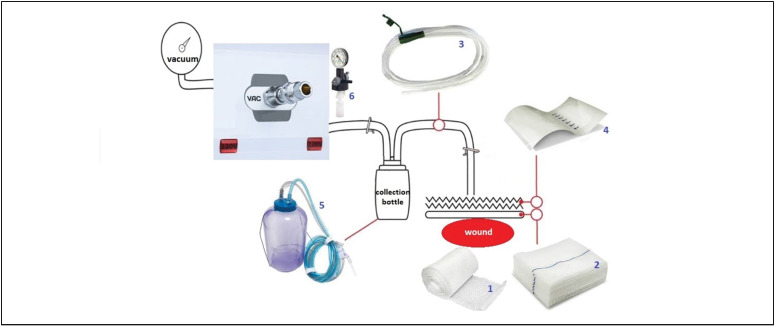
1: Gaze Rayon, 2: Dressing; 3: Gastric Tube Levine N 16, 4: Transparent adhesive film, 5: Suction bottle and filter 6: manometer (pneumatic), vacuum dressing model.

Source: Author.

The interventional procedures performed were wound treatment using skin grafts and at the same surgical time the application of vacuum dressing with cellulose acetate (Rayon) over the wound bed, sterile surgical dressing, 16 French nasogastric tube, sealed with a sterile transparent polyurethane film dressing connected to the secretion collection system with a backflow valve linked to the hospital vacuum meter with continuous pressure regulated to 100 to 120 mmHg. (Figure 1)

**Figure 2:**
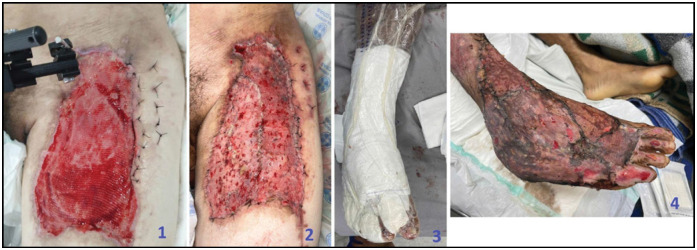
1: Morel-Lavallée injury post vacuum dressing 2: Morel-Lavallée injury post partial skin graft and vacuum. 3: Vacuum dressing mounted 4: Injury due to degloving (Partial skin graft + vacuum dressing after 5 days of procedure).

Source: Author.

The dressing was monitored in the ward during hospitalization to assess the pressure on the vacuum meter, bleeding, or detachment of the dressing due to shear or friction, as well as hematoma of the covered area. The dressing was removed for evaluation between the 5th and 7th day, a period in which the phases of graft take, plasma imbibition, inosculation, and revascularization normally occur. Partial thickness skin grafts are typically adherent after 5 to 7 days with the advancement of the wound healing stages. (Figure 2)^3^

After removal of the dressing with TPN, the graft was monitored weekly until complete healing. A questionnaire was developed for data collection with basic information on cause, type of trauma, age, as well as the assessment of the composition of the wound area post-graft, the percentage of tissue where shear, hematoma, or necrosis occurred at the time of vacuum removal. These factors were evaluated in a single stage by the same team. Since they are factors responsible for the failure of skin grafts and delay in the healing process. Data collection was performed during the postoperative follow-up.

## RESULTS

A total of 15 patients were included by the end of the study, with complete follow-up until total healing. The average age of the selected patients was 38.27 years. Regarding sex, we had 93.3% male and 6.7% female.

Only 26.7% of patients had pre-existing comorbidities. Of which only 1 (6.7%) had type 1 diabetes *mellitus*, which can hinder the healing process. The others presented hypertension, asthma, and schizophrenia.

Of the selected patients, 33.3% had active addictions at admission, with the most cited being the use of illicit drugs such as marijuana and cocaine, in addition to legal drugs like alcohol and tobacco.

In Table 1 we can observe that the main causes are traffic accidents with 60% of cases of major trauma with skin loss, requiring grafting. In addition to the location of these injuries, with 76.55% in the lower limbs.

**Table 1:** Type of trauma and location of the injury.

Type of Trauma		
	**N**	**%**
Accidents Motorcycle/Car/Truck/Train	9	60
Tumor	2	13.3
Runover	3	20
Injury + Osteomyelitis	1	6.7
Total	5	100
**Location of the injury**		
	N	%
Thigh	1	6.7
Leg	3	20
Leg and foot	3	20
Forearm	1	6.7
Arm	1	6.7
Hand	1	6.7
Foot	2	13.3
Popliteal fossa	1	6.7
Thigh, Knee, and Popliteal fossa	1	6.7
Thigh + Forearm	1	6.7
Total	15	100

Scope of the propositions and goals of the study demonstrating the applicability of low-cost TPN on skin grafting using the hospital gas meter vacuum, associated with routine hospital materials. Validation of the technique of using the vacuum meter and low-cost materials in the immediate postoperative of definitive closure of skin grafting in patients resulting from trauma.

**Figure 3:**
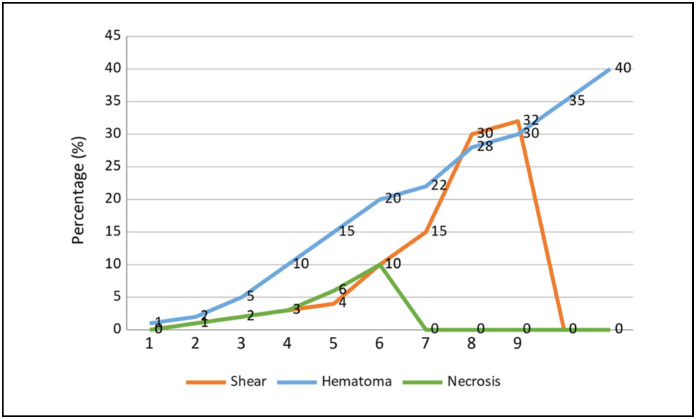
Size of the Lesion versus percentage of Shearing, Hematoma, and Necrosis.

Regarding the complication rate of grafts, we had a variance of 0 to 40%. The average hematoma was 15.73%, shear 8.07%, and necrosis 2.6%. It can be verified that in the use of TPN the lowest observed rate was necrosis. (Figure 3)

Regarding size, we had an average of 13.6 cm in length and 12.8 cm in width of the lesions, with the largest size being 38 cm and 33 cm respectively. At the moment we compared the size of the lesions with the percentage complication index, we noticed that the highest rates of hematoma, shearing, and necrosis occurred in patients with the largest area of skin loss and consequently with the most grafts performed.

## DISCUSSION

The average age of the patients was 38.27 years. The most affected age group is from 20 to 59 years, representing about 70% of deaths from traffic accidents. Young people between 21 and 30 years are particularly vulnerable due to greater exposure and risk behaviors in traffic, such as high speed and driving under the influence of alcohol.^6^

Men are the main victims, representing about 81% of deaths, corroborating our data. This predominance is attributed to risk behaviors, such as a higher frequency of aggressive driving and alcohol consumption before driving.^6^

The study data revealed that the lower limbs were more affected. The predominant injuries include trauma to the limbs, such as fractures and contusions, which are common among survivors.^6^

The cost of hospitalizations and productivity losses due to traffic accidents is estimated in billions of reais annually, highlighting the economic and social relevance of the problem.^6^

In the study by Souza et al.^7^ that evaluated the financial viability of TPN, participants were divided into two groups: wound treatment with the simplified vacuum dressing model (MCVS), which is the TPN model of our study, and the other group with hydrofiber treatment with silver (HFP). The participants in the study were mostly men with an average age in the 6th decade. Regarding age, the average in our study was 38.27 years.

In the study by Kamamoto,^4^ which is the precursor of low-cost TPN in Brazil, participants were also divided into two groups: TPN USP group and VAC (*Vacuum Assisted Closure*). Regarding trauma, the TPN group (94%) and VAC group (84%) had traffic accidents as the main cause, representing 60% of cases in our study. Regarding treatment costs, the TPN group had an average cost of R$47.89 and the VAC R$2,757.40. The VAC system uses polyurethane foam while TPN, both from USP and ours, uses rayon gauze and compress, as it is sterile, low-cost, highly available in health services, and less painful when changing the dressing. Regarding wound healing, satisfaction with the results comes from faster regeneration, with lower risks of contamination and complete closure of the lesion in less time compared to the VAC group.

According to the reviews by Santos et al.^8^ and Lima et al.^1^, TPN helps create a conducive environment for healing by stimulating granulation tissue and perfusion, as well as reducing edema and removing exudate and infectious material. In the postoperative period, it promotes tissue formation after debridement and reinforces skin grafts, favoring moisture balance, advancing the epithelial edge, and the rate of graft non-integration is lower when TPN is used. In line with this, TPN becomes an ally in graft integration.

According to Scalise et al.^9^, hematomas and seromas are complications resulting from the accumulation of blood and serum, respectively, in internal spaces. Even with excellent surgical technique, bleeding and inflammation can occur, and consequently, serum leakage increases the likelihood of infection, slower healing, additional clinical visits, and surgical interventions. In the study using low-cost vacuum dressings, even though the average hematoma rate was 15.73%, it did not contribute to loss or the need for new interventions on the graft.

Condé-Green et al.^10^ in their study compared the treatment of open abdominal wounds with traditional dressings and negative pressure therapy, which showed rates of skin and fat necrosis of 9% with TPN and 18% with traditional dressing, respectively.

The percentage of necrosis of the partial skin graft was evaluated in our study and presented a percentage of 2.6%. The low index correlates with the aforementioned study.

Alves et al.^11^ highlight that the most frequent causes of graft loss are the presence of hematoma, which mechanically separates the graft from its bed, and shear movements, which prevent graft adhesion, both hindering vascularization. Shear occurred in 8.07% of cases treated with graft plus negative pressure therapy.

For Webster et al.^12^, graft loss rates may be lower when negative pressure therapy is used. Products designed and built in hospitals are as effective in this area as commercial applications. There are clear cost benefits when non-commercial systems are used to create the negative pressure needed for wound therapy, with no evidence of a negative effect on clinical outcomes.

It was observed that, with the increase in the size of the wound area, shear rates, hematoma, and necrosis also increased. This fact can be explained by the difficulty of maintaining a homogeneous and continuous pressure over large graft areas.

More than half of the research participants were victims of automobile accidents, which generate multiple injuries, both soft tissue and bone, with indications for external fixation to control damage. During the application of TPN on grafts, one of the difficulties was creating a vacuum due to air entering the peri-fixator system. Thus, as described by Lima et al.^1^, it is necessary to cut the adhesive film dressing into smaller fragments to achieve sealing and correct vacuum loss, which can be assessed by listening for escape points.

The article by Gao et al.^13^ reinforces the data presented above. The authors demonstrated in their article that the healing time of the analyzed wounds was shorter compared to the control group, with a significantly lower healing rate.

## CONCLUSION

This study highlighted the reduction of hematoma, necrosis, and shear. It denoted the versatility and effectiveness of vacuum dressings for various wound sizes. It enabled the implementation of an effective and safe system that reduced the complications caused by traumatic wounds. In this context, the development of a wound repair technique that optimizes the healing of devitalized tissues was important.

It described available resources in everyday hospital settings and low-cost options to create a vacuum dressing aimed at reducing costs associated with the treatment of soft tissue injuries, in addition to being an adjunct in the repair of injuries with indications for skin grafting.

Thus, the results suggested that the use of low-cost TPN on skin grafts is a highly valuable tool, safe, easy to reproduce, and with low morbidity to the surgeons’ technique arsenal. It contributed to improving the adhesion of grafts to the recipient bed and subsequent healing of complex injuries that require skin coverage, often leading to prolonged hospitalization and difficult therapeutic management.

## Data Availability

The underlying contents of the research are available in the manuscript.
